# Intensity, dynamics and deficiencies of empathy in medical and non-medical students

**DOI:** 10.1186/s12909-021-02927-x

**Published:** 2021-09-10

**Authors:** Krzysztof Sobczak, Agata Zdun-Ryżewska, Agata Rudnik

**Affiliations:** 1 Department of Sociology of Medicine and Social Pathology, Faculty of Health Sciences, Medical University of Gdansk, Tuwima 15 Str., 80-210 Gdansk, Poland; 2 Department of Quality of Life Research, Faculty of Health Sciences, Medical University of Gdansk, Gdansk, Poland; 3 Institute of Psychology, University of Gdansk, Gdansk, Poland

**Keywords:** Empathy, Medical education, Medical student

## Abstract

**Background:**

Empathy is an important competence in the professional development of medical students. The purpose of our study was to compare the levels and scales of empathy in people studying in different educational strategies.

**Methods:**

The study was conducted between April 2019 and March 2020. Medicine, nursing, midwifery, physiotherapy, psychology, pedagogy and sociology students were the participants of this study. University students preparing for medical professions (*n* = 1001) and students of programs unrelated to medicine (*n* = 700) underwent the Empathy Quotient test (EQ-40). We have compared results in both study groups with the use of the distribution of density, analysis of variance and student’s t-test.

**Results:**

The average results received by students of the university preparing for medical professions were lower (M = 42.6) than those of the non-medical university students (M = 45.3) and the differences between the universities turned out to be statistically important (t = − 5.15, df = 1699, *p* < 0.001). As many as 14.6% of the students in the 1st EQ class were preparing for various medical professions while 9% studied social sciences. 18.2% of all medical programme students (*n* = 412) manifested the lowest empathy class. Our research has revealed that the students with Asperger profile (AP) and high-functioning autism (HFA) studied at universities preparing for medical professions (*n* = 18) more frequently than at non-medical universities (*n* = 5).

**Conclusions:**

We have noticed a serious indicator of erosion in the levels of empathy in medical students and an increase in the number of people with AP and HFA. Empathy decreases in students after the third year of their studies, regardless of the kind of university. We recommend an introduction of career counselling when specialization is being chosen.

## Background

Empathy is one of the basic competences of health professionals, which they can utilize to provide successful patient-centred and relationship-centred care [1]. Therefore, a high level of empathy is significant for the development of professionalism in students preparing for medical professions [2]. Many publications document the effectiveness of educational interventions which aim at maintaining or increasing the level of empathy [3–6]. At the same time, many reports point to the low emotional and cognitive empathy index in students who are preparing for medical professions [7, 8]. Importantly, some papers show an erosion of empathy in students preparing for medical professions [9, 10].

We ran a pilot study on a group of 536 Medical University of Gdansk students between November 2017 and June 2018 [11]. During the analysis of the distribution of levels of empathy, we observed that as students progress through the years of medical education, their levels of empathy decrease. Certain researchers seek explanation for this tendency in the clinical experience, that begins after the third year of education [12, 13]. We concluded that the findings of our research justify an attempt to verify such hypotheses, and that it is worthwhile to carry out a study focused on a comparison of the levels of empathy between medical and non-medical students.

Though the subject of empathy in students preparing for medical professions is documented extensively in literature, comparative studies are lacking. There is a significant deficiency of studies, which compare the level of empathy in individuals studying in bio-medical and non-medical fields. Therefore, we wanted to make a comparison of the levels of empathy in students preparing for medical professions and students of social sciences. We also wanted to determine the distribution of empathy in reference to particular fields of study, genders, time and type of education. Finally, we wanted to investigate how the scale and incidence of empathy disorders manifest themselves depending on the field of study.

## Methods

The main was conducted between April 2019 and March 2020. The project was completed based on a bilateral agreement between the universities and it was approved by the Ethics Committee for Research Projects of the Institute of Psychology at the University of Gdansk (4/2019). The students who consented willingly had a chance to participate in the anonymous study after their course classes.

We have used the Empathy Quotient questionnaire [14, 15] which was tested psychometrically and published in a Polish version [16, 17]. The EQ-40 test consists of 40 diagnostic questions in which the respondents use the four-point Likert scale. This tool was constructed for diagnosing the level of empathy in people with normal intelligence. We also added a separate card to the EQ test with 10 questions, which described the demographic and social situation of the participants. Those questions constituted independent variables, such as age, sex, year and field of study, learning mode, specialisation, employment during education and others. Further 3 questions concerned the self-esteem of the participants, pleasure resulting from helping others and the perception of own self-esteem. The participants were asked to fill a self-description questionnaire using a 10-point scale, where 1 was the lowest value and 10 the highest one. The questions referred to declarative and subjective impressions of the respondents. In case of self-report questions we did not analyse predictive validity.

The data was analysed with Statistica 13.1 software. For the numerical data, we used the mean and standard deviation analysis. Categorical data analysis was conducted through the distribution of density. We used the ANOVA variance analysis to observe the mean between the groups. Student’s t-test was used for comparing the means of the two groups. In the analysis of correlations between discontinuous variables and statistic heterogeneity of the groups we have used Pearson’s chi-square test. The value for *p* < 0.05 was assumed as statistically important.

## Results

1783 students of the Medical University of Gdańsk and the University of Gdańsk participated in the study. 82 people did not complete the test in full which is why the results were not taken into account. 1701 completed questionnaires were included in the final analyses, which equals to 95.4% of returned questionnaires.

### Empathy distribution analysis

The mean analysis in the distribution of empathy for the entire group revealed that the empathy quotient was 6 points higher in women. (Table 1).

**Table 1:** Characteristics of the respondents including EQ results

Participants	n (% of n)	Mean	Median	Minimum	Maximum	SD
Sex						
Female	1366 (81)	44.8	45	9	78	9.6
Male	319 (19)	38.9	39	14	84	10.2
Age						
18–20	634 (47)	44.1	45	13	84	9.8
21–23	825 (49)	43.8	44	12	78	9.9
> 24	236 (14)	42.1	42	9	71	10.3
Type and field of study						
Medical university	1001 (59)	42.6	43	9	78	10.1
Medical	414 (24)	42.3	43	14	78	10.8
Nursing	249 (15)	42.6	43	9	65	10.2
Midwifery	148 (8.7)	42.2	42	12	66	9.4
Physiotherapy	190 (11.2)	44.0	44	19	74	9.2
Non-medical university	700 (41)	45.2	45	13	84	9.6
Psychology	326 (19)	45.6	45	19	66	9.6
Sociology	140 (8)	42.7	43	17	84	10.5
Pedagogy	234 (14)	46.3	47	13	67	8.7
Year of study						
1st	472 (28)	44.8	45	13	84	10.1
2nd	323 (19)	43.3	43	15	74	9.4
3rd	292 (17)	44.2	45	12	71	10.5
4th	311 (18)	43.1	43	15	78	10.1
5th	297 (18)	42.7	43	9	69	9.8

With the use of the Student’s t-test (*t* = 9.8, *df* = 1683, *p* < 0.0001 for *n* = 1683) we have received statistically important differences in the levels of empathy between women (M = 44.8, SD = 9.62) and men (M = 38.8, SD = 10.23). It is in conformity with the psychometric principles/assumptions of the EQ-40 test in which women receive higher scores (*t* = 4.3, *df* = 196, *p* < 0.0001; means in the group of men M = 41.8 and women M = 47.2) [14]. The proportions between the number of female and male participants of the study closely resemble the proportions between the numbers of female and male students in each field. We did not observe indications of any overrepresentation. (Table 2).

**Table 2:** The number of students of each field including the percentage of female students

Field of study	Year of research	Number of students	Female students (%)	Number of participants*	Female participants (%)*
Medical	2019	1864	63.2	407	60.9
	2020	1945	62.4		
Nursing	2019	414	93.5	249	93.2
	2020	453	92.3		
Midwifery	2019	196	98.0	148	98.6
	2020	216	98.6		
Physiotherapy	2019	245	73.5	188	76
	2020	257	69.3		
Psychology	2019	643	82.6	323	82.7
	2020	672	83.8		
Sociology	2019	256	72.3	139	72.7
	2020	269	73.2		
Pedagogy	2019	889	96.4	231	99.1
	2020	901	96.6		

* The distribution of density applies to those participants, who declared their sex (*N* = 1685)

When we included the distribution of means in relation to gender and the kind of university, we received results which were lower than the ones that could be considered as normative. For a medical university, the mean empathy level for men was M = 38.5, SD = 10.3 and for women M = 43.8, SD = 9.8. In the case of degree courses in social sciences, we have discovered means which were closer to the anticipated ones which were M = 39.8, SD = 10 in men and M = 46.1, SD = 9.2 in women.

We have also observed a slight decline in the average empathy level with the increasing age of the respondents. The univariate analysis of variance ANOVA revealed statistically significant differences in the volume of empathy in particular years of studying in the entire study group (F = 2.56, *df* = 4, *p* = 0.037 for *n* = 1690). The post-hoc tests which were conducted revealed statistically significant differences between the first year (M = 44.71) and the fourth (M = 43.03, *p* = 0.02) and fifth year (M = 42.74, *p* = 0.008).

We have found discrepancies between the means of the students preparing for medical professions (M = 42.6, *SD* = 10.1 for *n* = 1001) and non-medical university students (M = 45.3, *SD* = 9.6 for *n* = 700). The confirmation of the statistical importance of the results was revealed with the use of the student’s t-test (*t* = − 5.15, *df* = 1699, *p* < 0.001 for *n* = 1699). The lowest results were received by the students of midwifery and the medical programme while the highest ones were received by the students of pedagogy and psychology. Taking into account the discussed distributions in relation to the degree courses and years of studies, we have noticed a correlation of the decrease in empathy only in the students who were preparing for medical professions. The most dynamic decrease was observed in students of physiotherapy. In the case of the students of social sciences, the fluctuation of the empathy level turned out to be inconclusive, apart from those studying pedagogy, in whom the average level of empathy increased (Table 3).

**Table 3:** Distribution of mean EQ for fields and years of studies

Filed od study	Year	N	Mean	Median	Minimum	Maximum	SD
Medical	1	81	43.1	43	14	67	11.6
	2	70	42.1	42	24	63	8.7
	3	69	43.1	44	20	66	11.0
	4	93	41.6	41	17	78	11.6
	5	99	41.8	43	14	64	10.7
Nursing	1	67	45.0	45	20	74	10.5
	2	33	45.6	46	19	68	8.7
	3	53	43.8	46	26	65	9.2
	4	47	41.0	39	24	61	7.8
	5	49	44.2	42	31	69	9.0
Midwifery	1	43	44.9	45	25	63	8.4
	2	37	40.7	41	15	57	9.7
	3	21	40.3	43	12	57	12.4
	4	24	41.4	40	27	66	9.5
	5	23	42.1	42	31	54	6.7
Physiotherapy	1	51	46.2	46	20	64	10.2
	2	36	40.7	40	27	63	9.3
	3	30	41.7	43	20	60	10.8
	4	33	41.5	42	15	62	9.8
	5	37	40.8	41	9	65	9.6
Psychology	1	96	47.8	49	13	66	9.0
	2	79	43.5	43	29	56	8.0
	3	69	48.0	48	22	67	9.5
	4	55	46.8	47	25	64	7.2
	5	27	44.3	44	23	65	8.7
Sociology	1	61	41.8	42	17	84	10.4
	2	32	45.1	43	23	74	10.5
	3	13	45.1	44	28	71	12.4
	4	20	42.7	42	20	59	9.2
	5	13	40.5	43	23	55	10.3
Pedagogy	1	73	44.7	44	26	66	9.1
	2	36	44.7	44	23	67	9.9
	3	37	46.5	47	27	69	9.1
	4	39	46.6	47	19	65	10.0
	5	49	46.5	44	28	66	10.6

### Empathy level disorders scale

According to the adopted division into empathy classes for the EQ-40 test, the score range for class I was 0–32. According to the theoretical assumptions, this class is represented by people with a low level of empathy or empathy disorders, most frequently affected by AP or HFA who get 20 points on average [16]. Class II represents the results between 33 and 51 points which translates into an average level of empathy in which most women get 47 points and men 42. In class III there are people with results between 52 and 63 points, which is above the average. Class IV is characteristic of people who have received 64–80 points, that is those with a very high or maximum EQ level [16].

When it comes to gender distribution, in class I there were 60.5% men (*n* = 127) and 39.5% women (*n* = 83). When we took into account the kind of university, we discovered that 14.6% of the students in class I were the students preparing for medical professions (*n* = 146) and 9% (*n* = 64) were social sciences’ students (Table 4).

**Table 4:** Association between year and field of study and empathy class

Filed od study	Class	n (% of n)					Total
		1st year	2nd year	3rd year	4th year	5th year	
Medical	I	13 (17.3)	8 (10.7)	11 (14.7)	22 (29.3)	21 (28)	75
	II	47 (18.4)	52 (20.4)	44 (17.3)	52 (20.4)	60 (23.5)	255
	III	19 (25.6)	10 (13.5)	11 (14.9)	17 (23)	17 (23)	74
	IV	2 (25)	0 (0)	3 (37.5)	2 (25)	1 (12.5)	8
Nursing	I	7 (18.9)	7 (18.9)	12 (32.4)	5 (13.6)	6 (16.2)	37
	II	39 (24.2)	21 (13.1)	29 (18)	34 (21.1)	38 (23.6)	161
	III	20 (40.8)	5 (10.2)	12 (24.5)	8 (16.3)	4 (8.2)	49
	IV	1 (50)	0 (0)	0 (0)	0 (0)	1 (50)	2
Midwifery	I	3 (15.8)	6 (31.6)	3 (15.8)	4 (21)	3 (15.8)	19
	II	30 (28.3)	26 (24.6)	15 (14.1)	17 (16)	18 (17)	106
	III	10 (45.5)	5 (22.7)	3 (13.6)	2 (9.1)	2 (9.1)	22
	IV	0 (0)	0 (0)	0 (0)	1 (100)	0 (0)	1
Physiotherapy	I	4 (26.6)	1 (6.7)	4 (26.7)	3 (20)	3 (20)	15
	II	37 (26.4)	29 (20.7)	20 (14.3)	28 (20)	26 (18.6)	140
	III	8 (29.6)	5 (18.5)	5 (18.5)	2 (7.4)	7 (26.0)	27
	IV	2 (40)	1 (20)	1 (20)	0 (0)	1 (20)	5
Psychology	I	10 (34.5)	8 (27.6)	6 (20.7)	4 (13.8)	1 (3.4)	29
	II	64 (31.2)	48 (23.4)	43 (21)	32 (15.6)	18 (8.8)	205
	III	21 (25)	20 (23.8)	18 (21.4)	18 (21.4)	7 (8.4)	84
	IV	1 (12.5)	3 (37.5)	2 (25)	1 (12.5)	1 (12.5)	8
Sociology	I	9 (45)	4 (20)	3 (15)	1 (5)	3 (15)	20
	II	45 (47.3)	19 (20)	7 (7.4)	15 (15.8)	9 (9.5)	95
	III	6 (28.6)	8 (38.1)	2 (9.5)	4 (19.1)	1 (4.8)	21
	IV	1 (33.33)	1 (33.33)	1 (33.33)	0 (0)	0 (0)	3
Pedagogy	I	3 (21.4)	5 (35.7)	2 (14.3)	1 (7.2)	3 (21.4)	14
	II	45 (28.8)	24 (15.4)	22 (14.1)	29 (18.6)	36 (23.1)	156
	III	24 (40.7)	7 (11.9)	11 (18.6)	8 (13.6)	9 (15.2)	59
	IV	1 (20)	0 (0)	2 (40)	1 (20)	1 (20)	5

The largest fraction of the students in class I were medical students. Exactly 75 students, or 18.2% of all the medical programme students (*n* = 412), received the result below 32 points in the EQ test. The proportion among nursing students was 14.8% and midwifery 12.8%. The lowest numbers of people with empathy disorders were found among pedagogy students (6%) and physiotherapy (8%) (Table 5).

**Table 5:** Distribution of people with class I EQ by fields and years of studies

Field of study	n (% of n)					Total (n)
	1st year	2nd year	3rd year	4th year	5th year	
Medical	13 (6.2)	8 (3.8)	11 (5.2)	22 (10.5)	21 (10)	75
Nursing	7 (3.3)	7 (3.3)	12 (5.7)	5 (2.4)	6 (2.9)	37
Midwifery	3 (1.4)	6 (3.0)	3 (1.4)	4 (1.9)	3 (1.4)	19
Physiotherapy	4 (1.9)	1 (0.5)	4 (1.9)	3 (1.4)	3 (1.4)	15
Psychology	10 (4.8)	8 (3.8)	6 (2.8)	4 (1.9)	1 (0.5)	29
Sociology	10 (4.8)	4 (1.9)	3 (1.4)	1 (0.5)	3 (1.4)	21
Pedagogy	3 (1.4)	5 (2.4)	2 (1)	1 (0.5)	3 (1.4)	14

In the course of our research we found 23 respondents who scored 20, or fewer, points in the EQ-40 test [16]. The largest number of people (*n* = 18) studied at the university which prepares for medical professions: 6 medical students, 6 students of nursing, 4 students of midwifery and 2 students of physiotherapy. We have noted 3 such people among sociology students as well as 1 among pedagogy students and 1 among psychology students.

We have received interesting results when we juxtaposed class I EQ with the values of self-esteem of the participants relating to perceived pleasure derived from helping others selflessly. People with the lowest levels of empathy who are studying to become medical professionals (*n* = 146) have declared a relatively high rate of satisfaction from helping others (M = 7.3, SD = 1.8) compared to the entire study group (M = 8.3, SD = 1.5 for *n* = 1698). When it comes to the analysis of the fields of study, the highest means in this category were observed among the pedagogy students (M = 8.7, SD = 1.4 for *n* = 234), midwifery (M = 8.6, SD = 1.3 for *n* = 148), and nursing (M = 8.6, SD = 1.3 for *n* = 249), and the lowest ones among sociology students (M = 7.9, SD = 1.5 for *n* = 139). A similar result was received after the analysis of the answers concerning the perceived self-esteem (M = 6, SD = 2.2) where for the whole group of respondents *n* = 1700 we have noted M = 6, SD = 1.9. We have calculated Pearson correlation coefficients for the subjective satisfaction derived from helping separately for the students studying to become medical professionals (*n* = 46) and students in the fields of study not related to medicine (*n* = 64) who represented class I EQ. We have noted correlations between empathy and the pleasure derived from helping which were not statistically significant (for medical majors *r* = 0.13, for non-medical majors *r* = − 0.005, *p* > 0.05).

## Discussion

Comparing the above data to the validation values of the EQ-40 test and the average results received by the students whose fields of study were not related to medicine (M = 45.2), it must be noted that the students preparing for medical professions were characterised by a slightly lower level of empathy. According to the psychometric qualities of the EQ-40 test, women received a higher average result when it came to empathy level than men, what is confirmed by many other reports [18–20]. The highest deviations in this aspect were noticed in the test results of the students training for medical professions where the difference in comparison to the validation values was −2.4 points for women and − 3 points for men. However, taking into account only the average empathy result at the university, which prepares for medical professions (M = 42.6), including medical programme (M = 42.3), the results turned out to be even lower than the results received in a corresponding studies by other authors. Haque et al.’s research revealed an average result in medical programme students of 36.7 [21], while Bangash et al. received the mean of 40.1 [22]. However, it must not be forgotten that EQ is characterised by high cross-cultural stability for Western countries, and is less stable and more susceptible to gender differences in Asian countries [23].

The results which were obtained were analysed from the diachronic perspective too. In 1989, in a corresponding study, Rembowski used Mehrabian and Epstein’s Emotional Maturity Scale (EMS) to analyse the level of empathy in medical students (*n* = 54) as well as students of psychology (*n* = 83) and pedagogy (*n* = 63) as well as technical fields of study (*n* = 52). The students of the same universities where we conducted our study as well (except for the technical university) received very different results. A high level of empathy was noted in 75% of all the participants. 24.6% of the respondents received an average result and only one psychology student received a low score (1.2%). In the medical programme only 64.8% of the students demonstrated a high level of empathy and 35.2% an average one [24]. In our study, the proportion of respondents who received a low score (class I) was 12.3%, average (class II) 66% and high or very high (class III and IV) 21.7%. Among medical students (*n* = 412), the low level was noted in 18.2% of the students, average in 61.9% and the high or very high level in 19.0% of the respondents. When comparing the results, we observed, that while students progress over consecutive years of medical education, their empathy levels decrease.

There are many reports in longitudinal studies which indicate a pattern of a decrease in empathy during medical education [25–28]. As some researchers suggest, this situation may result from the students’ professional socialization. In their opinion, a decrease in empathy, which comes with the beginning of practical training, may be connected with the widely understood clinical experience [29]. Other researchers associate the phenomenon of a decrease in empathy after the third year of studying with the students’ professional socialization which refers to the attitudes and behaviour of clinical teachers [12]. There also are reports, which indicate a connection between the decrease in the empathy level and the growing level of stress connected with the mode of teaching [13, 30] or a burnout resulting from the beginning of a clinical practice [31]. In our study, we have also observed a decrease in the level of empathy in medical students after the third year of studies. However, based on the juxtaposition of the means, we think that this phenomenon cannot be linked to the beginning of clinical apprenticeship. A decrease in empathy occurred at that time not only in medical students, physiotherapy and nursing students but also in students of sociology and psychology (Table 3).

It is also worth noting, that in his 1989 study, Rembowski found no cases of people with empathy disorders among the university students preparing for medical professions [24]. In our research, less than one-fifth of the medical students manifested a low level of empathy. Exactly 23 students who took part in the project scored 20 or fewer points in the EQ-40 test.

The assumption that the empathic attitude must be closely connected with an actual will to provide help aimed at bringing relief to the patient is one of the foundations of the clinical concept of empathy [32–34]. From the theoretical point of view, empathic actions are motivated by the satisfaction from providing help to those who need it [35]. To analyse the validity of the altruistic motivation, we asked the students, who participated in the study, about the level of their satisfaction in this respect. The results we have received indicate that the medical students who manifested the lowest level of empathy also declared a relatively high level of perceived satisfaction. This could show that satisfaction from helping others does not have to be closely connected with empathy. In our opinion, such a state of affairs might indicate a categorical shift in the understanding of the category of “satisfaction derived from helping others” and be connected with fulfilling the obligation to help others (which arises from deontology) then directly from the sense of satisfaction derived from helping others.

However, our findings indicate a tendency towards a decrease in the empathy levels in students preparing for medical professions and students whose fields of study do not relate to medicine over the last 30 years. It is worth noting that a low level of empathy occurs as a factor which is independent from a one’s relatively high perceived self-esteem. A corresponding tendency was pointed out in the meta-analyses by S. Konrath et al. which indicated a 40% decrease in the levels of empathy in schoolchildren over the last 30 years and, at the same time, an increase in the number of personalities with narcissistic tendencies [36]. In our opinion, the decrease in empathy may be much deeper in students who are preparing for medical professions. The dynamics of this process is most likely conditioned by social changes pointed out by S. Konrath et al. When narrowing the context down, it is worth noting the socialization in reference to the teaching content at the schools preparing for medical professions. There is ample evidence indicating the efficacy of the educational intervention programmes aiming at increasing the level of empathy in students [4–6]. Despite this, courses which increase the level of empathy have not become a part of the teaching cannon in schools preparing for medical professions. When we analysed the number of hours that students of medical sciences spend learning subjects such as psychology, medical sociology and medical ethics, we found that they constitute 1.7% (95 h) of all the obligatory classes for future doctors. We have also noticed that the overall number of obligatory classes in behavioural and social sciences for medical students has decreased over the 30 years [37]. Therefore, we think the teaching perspective focused exclusively on the biomedical education may contribute to the decrease in the students’ psychosocial competences during their studies. On the other hand, the teaching material in the curricula of the schools preparing for medical professions, due to their unique biological and engineering character, may be an interesting choice for the personalities in the decreased spectrum of empathy. This observation is also confirmed by the results of studies in which pharmacy students participated too. Among these students, as many as 21.2% received scores from the lowest range (class I) of the empathy level [11].

The pragmatic aspect of humanistic education in teaching of the future medical professionals should shape individuals to become professional scientists and sensitive humanists at the same time [38]. Numerous evaluations of educational interventions, whose purpose was to develop empathy and compassion for patients in students, have shown a high effectiveness in this respect [2, 6, 39]. From our point of view, an offer of occupational guidance should be an important element of reorganizing the education in schools preparing for medical professions. The diversity of specializations within medicine gives the students a variety of choices in the strategies of constructing their professional careers. There are specializations in which empathy is almost a basic work tool. However there also are some in which the contact with the patients is limited to the bare minimum or there is no such contact at all.

When we were formulating the results of our research, we had some objective limitations in mind which occurred during their organization and completion. The first problem was connected with the possibilities of a broad comparison between the results which we obtained and the ones presented by other authors. A vast majority of the studies concerning the level of empathy in the students of schools preparing for medical professions was completed with the use of the Jefferson Scale of Physician Empathy [40]. We also decided that using JSPE for studies with students outside the medical context is unjustified, due to the dedicated character of JSPE. This is why we used the EQ-40 test which, in our opinion, was a more adequate tool for the non-medical context which we wanted to include. However, it is also useful in the studies on empathy in medical students [21, 22, 41, 42].

Another limitation of the research may be the fact that there is a statistically significant, albeit small, difference in empathy between students of medical and non-medical faculties. The average results of students of both universities are in the same class II, which includes results from 33 to 51 points. This difference of a few points to the disadvantage of medical students seemed to us worth presenting to because it is confirmed in other studies and may be related to a certain, growing trend that is worth observing and counteracting it.

The lack of results concerning medical students of the sixth year is a significant limitation which may impact the interpretation of the received results directly. The stage of field research which we had planned was not completed due to the epidemiological situation connected with SARS-CoV-2. We have decided not to conduct the study in a remote form, as we thought the psychosocial situation in which the students have found themselves could impact the study results significantly. The lack of data concerning medical students of the 6th year did not allow us to show the full dynamics of the level of empathy or to define the scale of empathy disorders incidence in medical students. The epidemiological situation also had an impact on the differences in the numbers of students in the compared groups which prevented us from collecting the planned number of statements from students whose fields of study were not related to medicine.

## Conclusions

Students who were preparing for medical professions received lower scores when it came to the levels of empathy than those at non-medical universities. It must be noted that there is a serious indication of decreasing empathy levels in medical students over the years. We think there is an urgent need for the introduction of practical courses developing skills connected with emotion management, empathy and compassion towards others into the obligatory teaching canon in schools preparing for medical professions.

## Data Availability

The datasets used and/or analysed during the current study are available from the corresponding author on reasonable request.
